# A Novel Soft Bending Actuator Using Combined Positive and Negative Pressures

**DOI:** 10.3389/fbioe.2020.00472

**Published:** 2020-05-19

**Authors:** Mohammad Fatahillah, Namsoo Oh, Hugo Rodrigue

**Affiliations:** Soft Robotics Laboratory, School of Mechanical Engineering, Sungkyunkwan University, Suwon, South Korea

**Keywords:** PNP actuator, vacuum-based actuator, soft robotics, artificial muscles, fluidic actuators

## Abstract

Most soft pneumatic actuators for producing bending actuation have made use of either positive or negative pressure and adjusted their design in consequence. In the proposed paper, a novel soft bending actuator using combined positive and negative pressures (PNP) where the bending force of a negative pressure actuator and a positive pressure actuator is combined into a single actuating structure. This actuator is capable of producing a blocked force as high as 150 N at a combined positive pressure of 60 kPa and negative pressure of 60 kPa while still being able to produce large bending deformations. It was found that the equilibrium angle of PNP actuation is lower than using only negative pressure but that the actuator can produce larger forces at angles below the equilibrium angle of using positive pressure only. The actuator can use PNP actuation to produce large forces at lower bending angles and negative pressure actuation for producing large bending angles. This actuator was implemented in a soft robotic gripper capable of lifting large objects weighing up to 4 kg and a soft pinching gripper capable of holding a notebook weighing 1.85 kg by pinching it. The proposed actuator is capable of large forces and is versatile such that it is expected to be used in applications such as agriculture where many objects tend to be large and heavy yet require a delicate touch.

## Introduction

Soft robotic grippers have attracted significant attention from industry for grasping objects in unstructured environments due to their compliance and ability to grasp objects without requiring precise alignment. Particularly, soft pneumatic grippers have shown significant promise as they can conform themselves to the surface of the object and because the force produced by these actuators can easily be modulated through varying the applied pressure (Rus and Tolley, 2015; Mirvakili and Hunter, 2018; Shintake et al., 2018). However, the grasping capabilities of soft pneumatic grippers is limited by the bending force of the soft pneumatic actuators from which their fingers are made.

Fluidic elastomer actuators (FEAs) generally consist of a polymeric matrix with cavities that expand upon pressurization and whose expansion causes an asymmetric deformation of the actuator which results in a bending motion. This is generally done by having a strain differential across the thickness of the actuator causing the longitudinal expansion of the actuator to produce a bending deformation. This anisotropic expansion can be induced into single material actuators by using walls of different thicknesses or by using asymmetric wall shapes (Wakimoto et al., 2009; Gorissen et al., 2011). The addition of a strain-limiting layer on one side allows for better bending actuation performance through a larger strain differential (Ilievski et al., 2011). Adding a second constraint in the radial direction allows to focus the inflation in the longitudinal direction through the walls of the actuator not overinflating when pressurized. This radial constrain has generally been achieved through thicker wall thickness in the radial direction or by using fiber reinforcements either wrapped around the actuator or embedded within the matrix itself (Suzumori et al., 2007; Deimel and Brock, 2013; Galloway et al., 2013; Mosadegh et al., 2013; Polygerinos et al., 2013; Connolly et al., 2015). Using multiple cavities can also be used to realize bending in multiple directions (Suzumori et al., 1991; Martinez et al., 2013). These designs can also be realized through 3D printed polymers which allows for easier scaling down of the resolution or more complex designs (Schaffner et al., 2018; Vogt et al., 2018; Zhang et al., 2019). 3D printed thermoplastics have also been used to produce bending actuators capable of bending forces up to 80 N at a pressure of 200 kPa, the largest bending force of all of the surveyed pneumatic bending soft actuators (Yap et al., 2016).

The use of thin films or textiles instead of rubbers permits the fabrication of very lightweight actuators without necessarily lowering their performance in comparison with polymer-based actuators. Pouch motors with rigid constraints between pouches have been used to produce bending actuators (Niiyama et al., 2015; Oh et al., 2019). Tubes made with inextensible materials with folds have been used to realize smooth or jointed deformations using thermoplastic films (Nishioka et al., 2012, 2017; Amase et al., 2015; Sareen et al., 2017). Bending actuators using inflatable bellows and folded tubes have been developed capable of large forces and deformations (Best et al., 2015; Felt, 2019). The use of anisotropic textiles has been used to make bending actuators for wearable applications (Cappello et al., 2018).

Although all of the aforementioned actuators rely on a positive pressure differential with the environment, it is also possible to produce movement through a negative pressure differential with the environment (Yang et al., 2015, 2016, 2017; Robertson and Paik, 2017; Jiao et al., 2019; Lee and Rodrigue, 2019). Bending vacuum-based actuators have been realized using a rigid skeleton placed inside of a bladder (Li et al., 2017). Similar vacuum-based bending actuators have been realized used 3D printed structures with a measured maximum blocked force of 16 N (Tawk et al., 2018). However, all fluidic-based soft bending actuators have either used either positive or negative pressure.

The present work shows how both positive and negative pressures (PNP) can be used in tandem to produce greater bending forces and some of the limitations of doing so. A soft bending actuator combining PNP chambers into a single actuating structure where the force of both actuators is combined to produce large bending forces is presented in this paper. The effect of this design on both the bending angle and force of the actuator is demonstrated experimentally and compared with a simple quasi-static model. The design, manufacturing, and experimental results of the actuator are presented, and the performance of the actuator applied to a gripper for gripping large and heavy objects is presented. Notably, the actuator produced a maximum blocked bending force of 150 N, which is larger than any of the surveyed pneumatic soft bending actuators.

## Design and Fabrication

### Actuator Design

The proposed PNP actuator consists of a single structure containing two separate pneumatic actuators where the pressurization of one and/or the vacuuming of the other causes the actuator to bend. The simultaneous pressurization and vacuuming of the respective actuators cause the structure to produce a larger bending force over a certain bending range than simply actuating either. As this actuator contains two different actuators, its actuation properties will depend on the characteristics of the actuators used and their respective actuation pressures.

**Figure 1:**
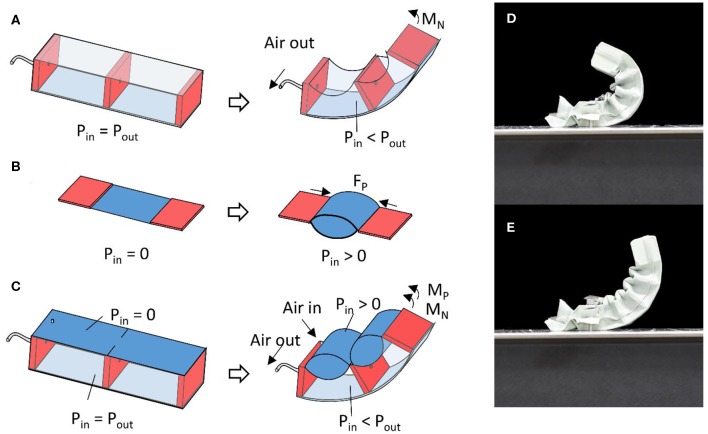
**(A)** Bending deformation of the actuator with only negative pressure actuation, **(B)** linear motion of a pouch motor, and **(C)** bending deformation of the actuator combining PNP actuation. Equilibrium of the actuator using **(D)** negative pressure actuation only and **(E)** PNP actuation.

The negative pressure actuator used in this work is composed of a sealed textile chamber containing a semi-rigid plate onto which rigid walls are attached. Vacuuming of the chamber causes the textile to push around the rigid walls and the semi-rigid plate to bend (Figure 1A). Although there are differences in geometry and materials, the base actuation mechanism is the same as previous works on vacuum-based bending actuators (Li et al., 2017; Tawk et al., 2018). The positive pressure actuator is a pouch motor located between the free ends of the rigid walls of the negative sub-actuator and its inflation causes a contractile force that pulls the free-end of the rigid walls toward each other that results in a bending motion of the semi-rigid plate in the same direction as that of the vacuuming of the negative pressure sub-actuator (Figure 1B). The pouch motor is similar to existing works in terms of function and materials, but its implementation into a bending actuator with a strain limiting layer differs from previous designs. The main differentiation with previous works is the use of both types of actuators in a single actuating structure. Simultaneous actuation of both positive and negative pressure chambers thus causes both actuators to create a bending force of the overall structure (Figure 1C).

It is to be noted that both actuators will likely have different equilibrium angles and that bending further than their respective equilibrium angle will cause this actuator to output a negative bending force. The negative bending force produced by either actuator will reduce the total bending force of the actuator as well as its maximum bending angle vs. using only the actuator with the highest bending angle. This can be seen in the proposed actuator by comparing the maximum bending angle using only negative pressure actuation and using PNP actuation (Figures 1D,E). The advantage of the proposed concept is the increased bending force produced at bending angles smaller than the equilibrium angle of the positive pressure actuator. However, it is still possible to use only negative pressure actuation for reaching large bending angle.

### Fabrication

**Figure 2:**
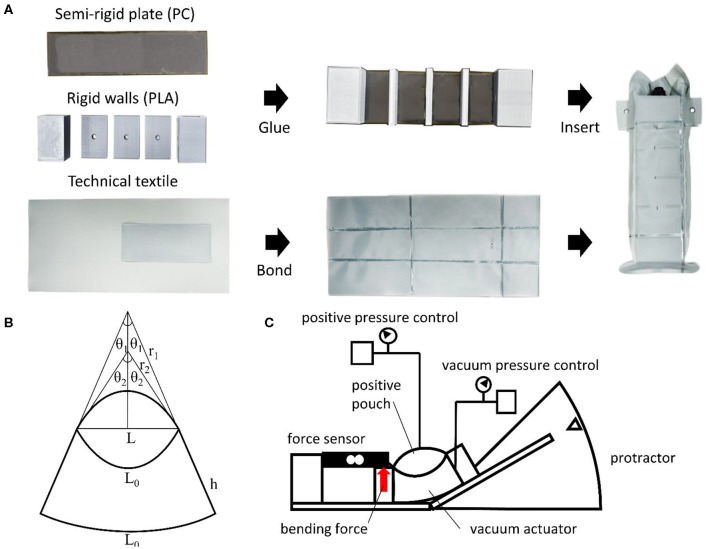
**(A)** Assembly process of the actuator, **(B)** dimensions of the actuator used for modeling, and **(C)** testing jig for measuring the bending force at different bending angles.

In the actuators presented in this work, the semi-rigid plate serving as the base of the actuator is manufactured from a 3 mm thick polycarbonate (PC) sheet which is cut into a rectangular shape using a laser cutter. The rigid walls are 3D printed using polylactic acid (PLA) filament and glued onto the PC sheet using superglue (Loctite 401, Henkel) (Figure 2A).

The sealed chambers of the actuator are made from a technical textile consisting of a one-sided thermoplastic polyurethane (TPU)-coated nylon fabric. Three sheets of textile are used in total where two are used for the upper and lower surfaces of the actuator and the third is for forming the pouch motors by bonding it to the inside portion of the upper textile sheet using an impulse sealer. The upper and lower surfaces are then bonded to form the negative chamber into which the plate and walls assembly is inserted.

### Modeling

The principle of conservation of energy will be used to produce a quasi-static model of the proposed PNP actuator. It is assumed that the film does not store any elastic energy and that the semi-rigid plate does not require any force to deform. This can be expressed as follows.

(1) $$\begin{array}{l} dW_{in}\;=\;dW_{out} \end{array}$$

Where the input work *W*_*in*_ is the work of fluid added or removed from the actuator and *W*_*out*_ of the output mechanical work of the actuator. The work of the fluid can be divided into the work of the negative and positive pressure actuators as follows.

(2) $$\begin{array}{l} P_{N}dV_{N}+\;P_{P}dV_{P}\;=\;Md\phi =Fld\phi \end{array}$$

Where *P*_*N*_ and *P*_*P*_ are, respectively, the pressures of the negative and positive pressure actuators, *V*_*N*_ and *V*_*P*_ are, respectively, the volumes of the negative and positive pressure actuators, *M* is the moment produced by the actuator and ϕ is the bending angle of the actuator (Figure 2B). This moment can be converted into the bending force *F* based on the moment arm *l* of the actuator, which is equal to the length *L*_0_ of the actuator when the force is applied at its tip. All pressures are gauge pressures. Assuming that the base of the actuator deforms with a constant radius of curvature throughout the motion we can obtain the following equations for the negative pressure sub-actuator.

(3) $$\begin{array}{l} L_{0}=2\theta_{1}\left(r_{1}+h\right) \end{array}$$

(4) $$\begin{array}{l} r_{1}\sin\theta_{1}=\frac{L}{2} \end{array}$$

Where *L*_0_ is the length of the semi-rigid plate forming the base of the actuator, *r*_1_ is the radius of the curve of the negative pressure sub-actuator, θ_1_ is the central angle of the negative pressure actuator also equal to half of ϕ, *h* is the height of the rigid walls of the actuator and *L* is the current length between the free-ends of the rigid walls. We can then obtain the following equations for the positive pressure sub-actuator.

(5) $$\begin{array}{l} L_{0}=2\theta_{2}r_{2} \end{array}$$

(6) $$\begin{array}{l} r_{2}\sin\theta_{2}=\frac{L}{2} \end{array}$$

Where *r*_2_ is the radius of the curve of the positive pressure actuator and θ_2_ its central angle. The relation between θ_1_ and θ_2_ can then be obtained from equations (4) and (6) as follows.

(7) $$\begin{array}{l} 2\sin\theta_{1}\left(\frac{1}{2\theta_{1}}-\frac{h}{L_{0}}\right)=\;\frac{\sin\theta_{2}}{\theta_{2}} \end{array}$$

This equation can be solved numerically or by using an approximation such as the Taylor series expansion. The area *A*_*p*_ of the positive pressure sub-actuator can be approximated as an airfoil shape with cylindrical surfaces as done in previous works on pouch motors.

(8) $$\begin{array}{l} A_{P}\;=\;\frac{L_{0}^{2}}{2}\left(\frac{\theta_{2}-\cos\;\theta_{2}\;\sin\;\theta_{2}}{\theta_{2}^{2}}\right) \end{array}$$

The area *A*_*n*_ of the negative pressure sub-actuator can be approximated as the sector formed by the line of the rigid walls minus half of the airfoil shape of the positive pressure sub-actuator and minus the triangular formed by the center of the circle of the sector and the free-ends of the rigid walls.

(9) $$\begin{array}{l} A_{N}\;=\;\frac{L_{0}^{2}}{4\theta_{1}}-\left[{\left(\frac{L_{0}}{2\theta_{1}}-h\right)}^{2}\left(\frac{\sin{2\theta }_{1}}{2}\right)\right]-\left[\frac{{L_{0}}^{2}\left(2\theta_{2}-\sin{2\theta }_{2}\right)}{8\theta_{2}^{2}}\;\right] \end{array}$$

The volumes can then be obtained by multiplying equations (8) and (9) by the depth *D* of the actuator as follows.

(10) $$\begin{array}{l} V_{P}=\frac{L_{0}^{2}D}{2}\left(\frac{\theta_{2}-\cos\;\theta_{2}\;\sin\;\theta_{2}}{\theta_{2}^{2}}\right) \end{array}$$

(11) $$\begin{array}{l} V_{N}=\frac{L_{0}^{2}D}{4\theta_{1}}-\;\left[{\left(\frac{L_{0}}{2\theta_{1}}-h\right)}^{2}\left(\frac{\sin{2\theta }_{1}}{2}\right)\right]D\\ \;-\left[\frac{{L_{0}}^{2}\left(2\theta_{2}-\sin{2\theta }_{2}\right)}{8\theta_{2}^{2}}\right]D \end{array}$$

These two equations together with equation (7) can then be differentiated and inserted into equation (2), but this results in a long formula that is omitted for brevity. As was done in this paper, it is also possible to use simple numerical methods such as finite difference approximation to evaluate these derivatives and evaluate the bending force produced by the actuator as a function of the bending angle.

Some simplifications of the geometry were made in terms of the chambers having a constant cross-sectional area throughout its width, which would not hold true toward the edges of the actuator for either of the chambers. The model predicts that the bending force produced toward zero bending angle is infinity for negative pressures. This is not feasible in practice as it assumes ideal unstretchable materials and that absolutely no manufacturing errors were made. Even a slack of 1–2 mm in the film would significantly affect the force at zero bending angle. It would be possible to develop a dynamic model of the actuator to predict its speed and frequency response, but this model would have to consider the dynamics of the pneumatic systems including its pump, regulators and valves as well as any drag forces induced by the pneumatic tubing.

### Method

A testing jig was manufactured to measure the bending force produced by the actuator at different bending angles (Figure 2C), and the bending force was measured using a load cell (CB1-K50, DaCell). The pressures within the positive and negative chambers were controlled using an electro-pneumatic regulator (ITV-1030, SMC) and an electronic vacuum regulator (ITV-2090, SMC), respectively. All experiments with error bars were repeated three times with the same actuator.

## Model Validation

**Figure 3:**
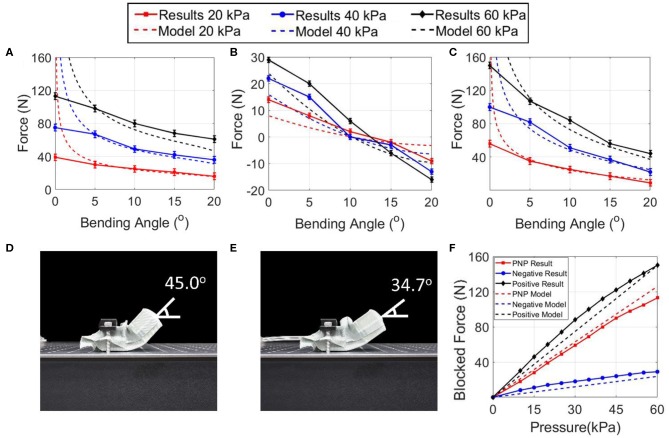
Bending force vs. bending angle at different pressures for **(A)** negative pressure actuation, **(B)** positive pressure actuation, and **(C)** PNP actuation. Equilibrium angle of the actuator with a single segment using **(D)** negative pressure actuation, and **(E)** PNP actuation. **(F)** Blocked force of the actuator vs. pressure.

An actuator with a single actuator chamber with dimensions of 30 mm in length, 80 mm in width and 30 mm in height was built and installed on the testing jig and connected to the electro-pneumatic regulators. Its bending force produced using either only negative or positive pressure was measured for pressures ranging from 20 to 60 kPa in increments of 20 kPa. The bending force produced by the actuator using only negative pressure at low angles does not reach infinity due to stretchability of the material and manufacturing errors, but the bending force produced by the actuator follows well the predicted trend throughout most of the range of angles (Figure 3A). The bending force produced by the actuator using only positive pressure exceeds that predicted by the model, but the equilibrium angle of the model and the experimental results are in general agreement (Figure 3B). The difference in bending force could be due to the assumption made that the cross-section of the actuator is constant throughout and due to manufacturing errors. The bending force using both negative and positive pressure for equal pressures from 20 to 60 kPa in increments of 20 kPa was then measured (Figure 3C). This experiment was conducted such that when the positive pressure actuator has a positive pressure of 20 kPa then the negative pressure actuator has a negative pressure of 20 kPa. The actuator follows well the predicted trend and appears to behave as the sum of the bending forces of the negative and positive pressure-only bending forces as predicted by the model.

An important value that can be observed to be predicted accurately by the model is the transition angle from PNP actuation producing a higher bending force to negative pressure-only actuation producing a higher bending force. This angle can be measured at the angle where negative pressure and PNP actuation produce an equal bending force. This value is important as it becomes preferable to use negative pressure actuation over PNP actuation at bending angles exceeding it. This value was measured to be equal to ~10° based on the values obtained experimentally for negative pressure and PNP actuation at all three tested pressure (Figures 3A,C) while the predicted value was 10.22° according to the numerical model. This value is for an actuator with a single segment, but an actuator with multiple segments would have a multiple of this value for the transition angle.

Next, the equilibrium angle of the actuator was measured for negative pressure and PNP actuation. This angle corresponds to the bending angle reached by the actuator at zero blocking force. This equilibrium angle was measured visually by using a pressure of 40 kPa for negative pressure actuation and with pressures of 40 kPa for both the positive and negative pressures using PNP actuation (Figures 3D,E). The measured equilibrium angles were 45.0° when using negative pressure actuation and 34.7° when using PNP actuation. This means that below 10° PNP is preferable, between 10 and 34.7° negative pressure is preferable and that between 34.7 and 45° only negative pressure is able to produce a bending force.

The blocked force of the actuator for positive, negative and PNP actuation was tested in the horizontal position up to 60 kPa (Figure 3F). It can be seen that the maximum force produced using only negative pressure was measured to be 113 N, using only positive pressure to be 29 N and using PNP to be 150 N. The force produced in all three modes was proportional to the input pressures, and the force achieved by PNP actuation is equivalent to that using only negative pressure plus using only positive pressure. With this configuration the use of PNP actuation produced an increase in blocked force of ~35%, which is substantial. The maximum blocked force achieved by the actuator is nearly double that of the highest blocked force produced by the surveyed pneumatic soft bending actuators. It is also to be noted that it takes longer for the actuator to reach a negative pressure of 60 kPa than it does to reach a positive pressure of 60 kPa due both to the larger volume of the vacuum portion of the actuator and the characteristics of pneumatic pumps.

## PNP Soft Gripper

Although soft grippers are quite adept at gently grasping small items that fit into the palm of a hand, few soft grippers have focused on grasping large objects whose weight may range up to a few kilos. This category of objects represents a sizeable portion of the objects found around us. A large gripper with four antagonistic fingers placed antagonistically with a total width of 270 mm was built using four PNP actuators with five segments and a total length of 144 mm each, a width of 50 mm and a height of 30 mm.

**Figure 4:**
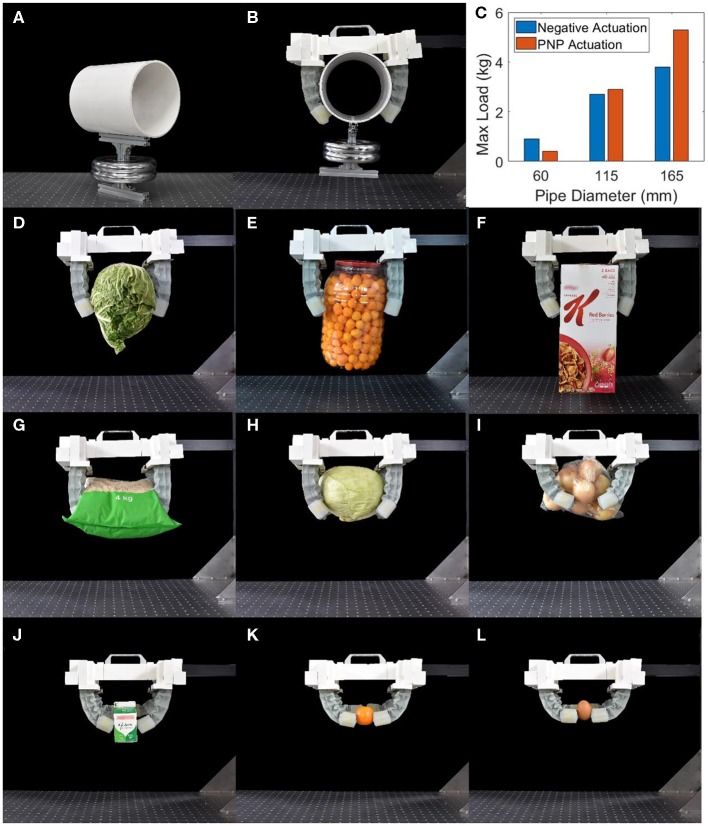
**(A)** Testing jig for maximum load vs. pipe diameter experiment (165 mm diameter pipe pictured), **(B)** gripper grapping the jig using PNP actuation, and **(C)** results for the maximum load for different pipe diameters. The gripper using PNP actuation for grasping **(D)** a head of cabbage (2.20 kg), **(E)** a large jar of cheese puffs (0.55 kg), **(F)** a large-sized box of cereals (1.30 kg), **(G)** a bag of rice (4.00 kg), **(H)** a head of cabbage (2.00 kg), and **(I)** a bag of onions (1.20 kg). The gripper using negative pressure actuation to grab **(J)** a carton of milk (0.28 kg), **(K)** a tangerine (0.06 kg), and **(L)** a boiled egg (0.04 kg).

It was seen previously that PNP actuation produces larger bending forces at lower bending angles while using only negative pressure for actuation results in a larger maximum bending angle. Considering this, it can be expected that PNP actuation would be preferable for grasping larger objects where a smaller bending angle is required to make contact with the object whereas using only negative pressure for actuation would be preferable for smaller objects where a larger bending angle is required for actuation. The maximum payload of the gripper for either negative or PNP actuation was tested for cylindrical jigs where the weight of the jig can be adjusted. Cylindrical jigs with external diameters of 60, 115 and 165 mm were tested with a negative pressure of 20 kPa for both cases and a positive pressure of 60 kPa for PNP actuation (Figures 4A,B). These values of the pressure were chosen as they are the pressures achieved by the actuator after ~1 s of actuation using a portable pump due to the negative pressure chamber being larger than the positive pressure chamber and due to the flow of air being larger for positive pressure. Results show that the gripper is capable of lifting much larger loads when using PNP for larger objects but that it is preferable to use negative pressure actuation for smaller objects (Figure 4C). Either type of actuation is suitable for medium-sized objects.

As the intended application of the proposed gripper is to grab larger and heavier objects found around us, the gripper was given some of the larger and heavier objects that can be found in a grocery store including a head of lettuce, a large jar of cheese puffs, a large-sized box of cereals, a bag of rice, a head of cabbage and a bag of onions (Figures 4D–I, Supplementary Video). The heaviest of these objects weighs 4 kg while others are large, slippery or cannot be grasped from the bottom. Smaller objects such as a carton of milk, a tangerine and a boiled egg were then grasped using only negative pressure by using the fingertips of the gripper (Figures 4J–L).

**Figure 5:**
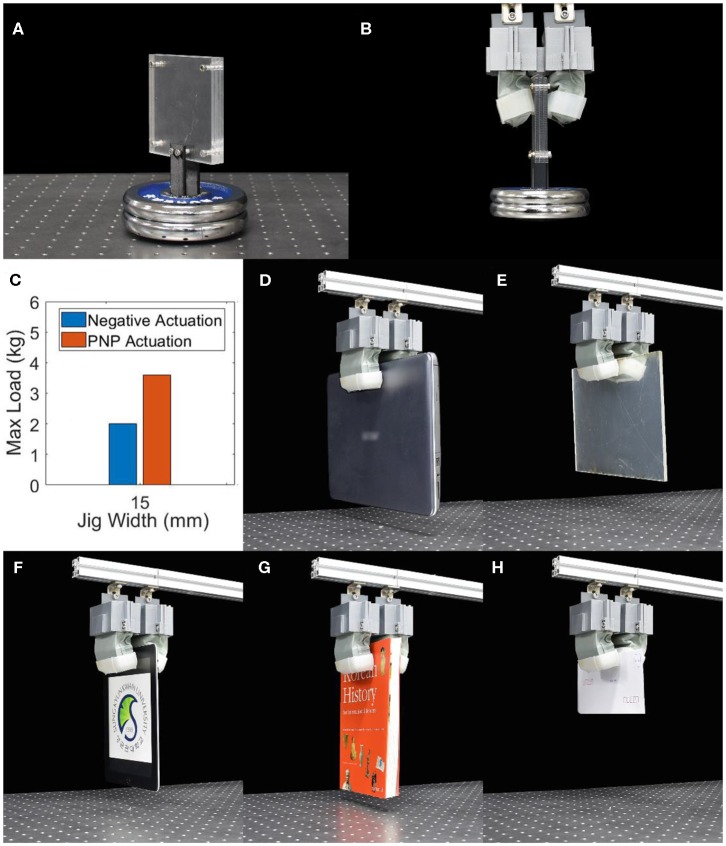
**(A)** Jig for testing of soft pinch gripper, **(B)** soft pinching gripper grasping this jig, and **(C)** maximum load for the soft pinching gripper using PNP actuation and negative pressure actuation. The soft pinching gripper grasping **(D)** a notebook (1.85 kg), **(E)** an acrylic plate (0.40 kg), **(F)** an electronic tablet (0.66 kg), **(G)** a book (0.78 kg), and **(H)** an envelope (0.01 kg).

A second gripper was built for pinching applications when grabbing large and slender objects which cannot be power grasped yet require large normal forces to produce the frictional force required to hold these objects. This soft pinching gripper consists of two antagonistic finger each with a single chamber with a width of 80 mm, a height of 30 mm, a length of 30 mm and a gap between the fingers of 15 mm. With the same pressures as the previous gripper, the equilibrium angle for negative pressure actuation was measured to be 45.0° and 34.7° for PNP actuation. The maximum weight held at these pressures by the gripper on a flat jig with a thickness of 15 mm made from acrylic plates was measured to be 2.0 kg for negative pressure actuation and 3.6 kg for PNP actuation, which represents an increase of 80% in the maximum payload (Figures 5A–C). The gripper was then made to grab flat and wide objects that cannot be grasped using a power grasp such as a notebook, an acrylic plate, an electronic tablet, a book and an envelope (Figures 5D–H). Weighing 1.85 kg, the notebook is the heaviest of these objects and is an object that most soft robotic grippers would struggle to successfully grasp and hold due to its large size and slippery surface.

## Discussion

Most soft pneumatic actuators use positive pressure for actuation and a few recent actuators have used negative pressure for actuation. This is the first actuator where a combination of both simultaneous positive and negative pressure can be used in a single actuator to create a larger bending force. It was seen that both positive and negative pressure actuation can contribute to the force produced by the actuator. The actuator's range of motion can be reduced since the actuator is a combination of two separate structures with different equilibrium angles such that, in the case of the design presented in this work, the positive pressure structure creates a negative bending force above its equilibrium angle. This is specific to the design presented in this work and the use of different dimensions could make both positive and negative pressure structures have equal equilibrium angles. A different design could perhaps make the equilibrium angle for both positive and negative pressures equal regardless of the dimensions of the actuator.

This reduction in the maximum bending angle might an issue when trying to grab objects requiring large deformations of the fingers, but the addition of positive pressure is not necessary to drive the actuator such that positive pressure actuation can be used selectively when trying to grab larger and heavier objects. An increase of 35% in the maximum blocked force was measured for PNP vs. using only negative pressure, but this was measured by using equal values of negative and positive pressures. Negative pressure is limited in pressure due to the limit between a perfect vacuum and room pressure while positive pressure can be increased as high as the structure can withstand. Future version of the actuator may be able to hold much higher pressures than those shown in this work such that the increase in blocked force may be much larger.

When used to grab objects using either power grasping or pinch grasping, the actuator is capable of outputting very large bending forces using PNP actuation at lower bending angles that can be used to grab larger and heavier objects. Negative pressure actuation can be used to grab smaller and lighter objects at higher bending angles. The design can also be used to build pinching grippers that can grab flat and wide objects that might require large blocked forces to produce enough frictional forces to prevent the object from slipping. The range of objects grasped in this work in terms of dimensions, shapes and weight is quite different from previous works focusing on smaller objects that can fit in the palm of a human hand and shows how soft robotic actuators can be also be used for objects that have not traditionally been linked with soft robotics. It is expected that this kind of gripper could be used in agriculture where some vegetables are large and heavy while still requiring a gentle touch to not damage them.

## Conclusion and Future Work

A novel design for a pneumatic soft bending actuator able to actuate using both positive and negative pressure was presented in this work. The main novelty of this work consists of using both positive and negative pressure for soft actuation which was demonstrated to be able to increase significantly the blocked force of the actuator. The maximum blocked force of the actuator measured in the work was ~150 N, which is larger than any of the other surveyed pneumatic soft bending actuators. This blocked force was achieved at the relatively low pressures of 60 kPa of positive pressure and 60 kPa of negative pressure. It was seen that the maximum bending angle of the actuator was reduced when PNP actuation was used, and that the increase in bending force is only for a given range of actuation. Above this point, using only negative pressure results in higher bending forces and larger bending angles. The actuator can be used in both modes of actuation and can thus benefit both from large forces at smaller bending angles and from larger maximum bending angles.

The proposed actuator was used to form two types of soft gripper. The first is a large power grasping gripper for larger objects that are often heavier than smaller ones but that can still grab smaller objects using negative pressure actuation. The second is a pinch grasping gripper meant to grab flat and wide objects requiring large blocked forces to produce sufficient friction force and prevent the object from slipping. Future work will focus on reinforcing the construction of the actuator to increase the maximum positive pressure held by the actuator and on further improvements to the design as well as testing the gripper for agricultural applications.

## Data Availability Statement

The raw data supporting the conclusions of this article will be made available by the authors, without undue reservation, to any qualified researcher.

## Author Contributions

MF and NO developed the experimental setups and conducted all experiments. MF and HR developed the numerical model, carried out the data analysis, and wrote the manuscript.

## Conflict of Interest

The authors declare that the research was conducted in the absence of any commercial or financial relationships that could be construed as a potential conflict of interest.

## Supplementary Material

Supplementary Video PNP soft robotic gripper grasping various objects.
